# Retinal and choroidal thickness in pediatric patients with sickle cell disease: a cross-sectional cohort study

**DOI:** 10.1186/s40942-021-00351-3

**Published:** 2022-03-04

**Authors:** Juliana Prazeres, Luiz Filipe Lucatto, Adriano Ferreira, Nilva Moraes, Josefina A. P. Braga, Luiz H. Lima, Caio Regatieri, Maurício Maia

**Affiliations:** 1 Department of Ophthalmology, Federal University of São Paulo, 806, Botucatu Street, São Paulo, 04026-062 Brazil; 2 Department of Pediatrics, Federal University of São Paulo, São Paulo, Brazil

**Keywords:** Retinal thickness, Choroidal thickness, Sickle cell disease

## Abstract

**Background:**

To measure the retinal/choroidal thicknesses in the macular area of asymptomatic pediatric patients with sickle cell disease (SCD).

**Methods:**

This cross-sectional cohort study included 40 children (79 eyes) with SCD and 19 control patients (36 eyes). All subjects underwent spectral-domain optical coherence tomography (SD-OCT) with enhanced-depth imaging OCT. Generalized Estimating Equations (GEE) were applied to compare the outcomes between groups. P ≤ 0.05 was considered significant.

**Results:**

The choroidal thickness in the macular area in the study subfields was significantly thinner in the SCD eyes compared with control eyes (subfoveal subfield and temporal parafoveal subfield, p < 0.0001; nasal parafoveal subfield, p < 0.0001 temporal perifoveal subfield, p < 0.0001; and nasal perifoveal subfield, p < 0.0001). The variations in the retinal thickness were not significant.

**Conclusion:**

EDI-OCT showed that the macular choroidal thickness is thinner in asymptomatic pediatric patients with SCD.

## Background

Sickle cell disease (SCD) is an autosomal recessive hematologic disease that results from the substitution of glutamic acid by valine in the hemoglobin molecule [1]. Consequently, the hemoglobin solubility changes resulting in chronic hemolysis and vaso-occlusive events. The irregular shape of the red blood cells may lead to blood flow disturbances, chronic hemolytic anemia, and several systemic complications [2].

Visual loss has been reported in 10% to 20% of patients with SCD possibly due to clinical manifestations in several parts of the eye, including the conjunctiva, iris, uvea, and retina [1]. A higher prevalence of retinopathy is seen in patients with hemoglobin SC compared to those with hemoglobin SS. Although the retinal findings in SCD are predominantly in the retinal periphery, macular changes also can occur including macular infarctions and enlargement of the foveal avascular zone with perifoveal capillary dropout [3, 4].

Previous studies in adults with SCD using spectral-domain optical coherence tomography (SD-OCT) have reported retinal thinning in the macular area due to retinal arteriolar occlusion and decreased choroidal thickness [5–8]. Although the macular findings in adults with SCD have been well described, few studies have specifically assessed the macula in pediatric patients [9, 10]. These reports showed retinal atrophy with macular thinning and flow voids in both the superficial and deep retinal capillary plexus. The purpose of this study was to analyze the retinal and choroidal thickness measurements in the macular area of asymptomatic pediatric patients with SCD using SD-OCT. To the best of our knowledge, this is the first study to evaluate the choroidal thickness in pediatric patients with SCD.

## Methods

The institutional review board and ethics committee of Federal University of São Paulo, São Paulo, Brazil, approved this study (Ethics Committee Number, 1529/11), which complied with the tenets of the Declaration of Helsinki.

A retrospective analysis was performed in consecutive asymptomatic pediatric patients with SCD seen in the Department of Ophthalmology of the Federal University of São Paulo, São Paulo, Brazil. The diagnosis of SCD was confirmed by hemoglobin electrophoresis. Patients were excluded who had a best-corrected visual acuity (VA) below 20/20, clinical evidence of maculopathy, epiretinal membranes, a spherical equivalent refractive error exceeding 6 diopters (D) of myopia, amblyopia, lens or vitreous opacity, retinopathy of prematurity, or any systemic condition such as diabetes mellitus, systemic hypertension, or an infectious or autoimmune disease that can cause retinal vascular disease.

All study patients underwent VA measurement using the Snellen chart, slit-lamp biomicroscopy, indirect ophthalmoscopy after pupil dilatation, and fundus photograph (Visucam, Carl Zeiss Meditec, Jena, Germany). The SD-OCT retinal and choroidal images were obtained using the Heidelberg Spectralis (version 1.5.12.0, Heidelberg Engineering, Heidelberg, Germany). Each OCT section was comprised of 100 averaged scans acquired using eye tracking that were taken in a 5 × 30-degree rectangle including the optic nerve and macula. In addition to conventional OCT scans, the choroid was imaged with the Heidelberg Spectralis using enhanced-depth imaging (EDI). All OCT scans were obtained between 9:00 and 11:00 am. Two examiners performed the measurements independently, and if the difference between their thickness measurements was higher than 15% of the mean of two values, a senior author was consulted.

The retinal measurements were performed according to the thickness map with nine Early Treatment Diabetic Retinopathy Study (ETDRS)-like subfields displayed. On the ETDRS map, the macula is divided into nine regions with three concentric rings measuring 1 mm (innermost ring), 3 mm (inner ring), and 6 mm in diameter (outer ring) centered on the fovea. The 1-mm innermost ring is the fovea, and the inner 3-mm ring and outer 6-mm ring were divided further into four equal regions, i.e., the central, parafoveal and perifoveal superior, temporal, inferior, and nasal subfields. The macular volume was recorded from the retinal thickness map.

**Fig. 1:**
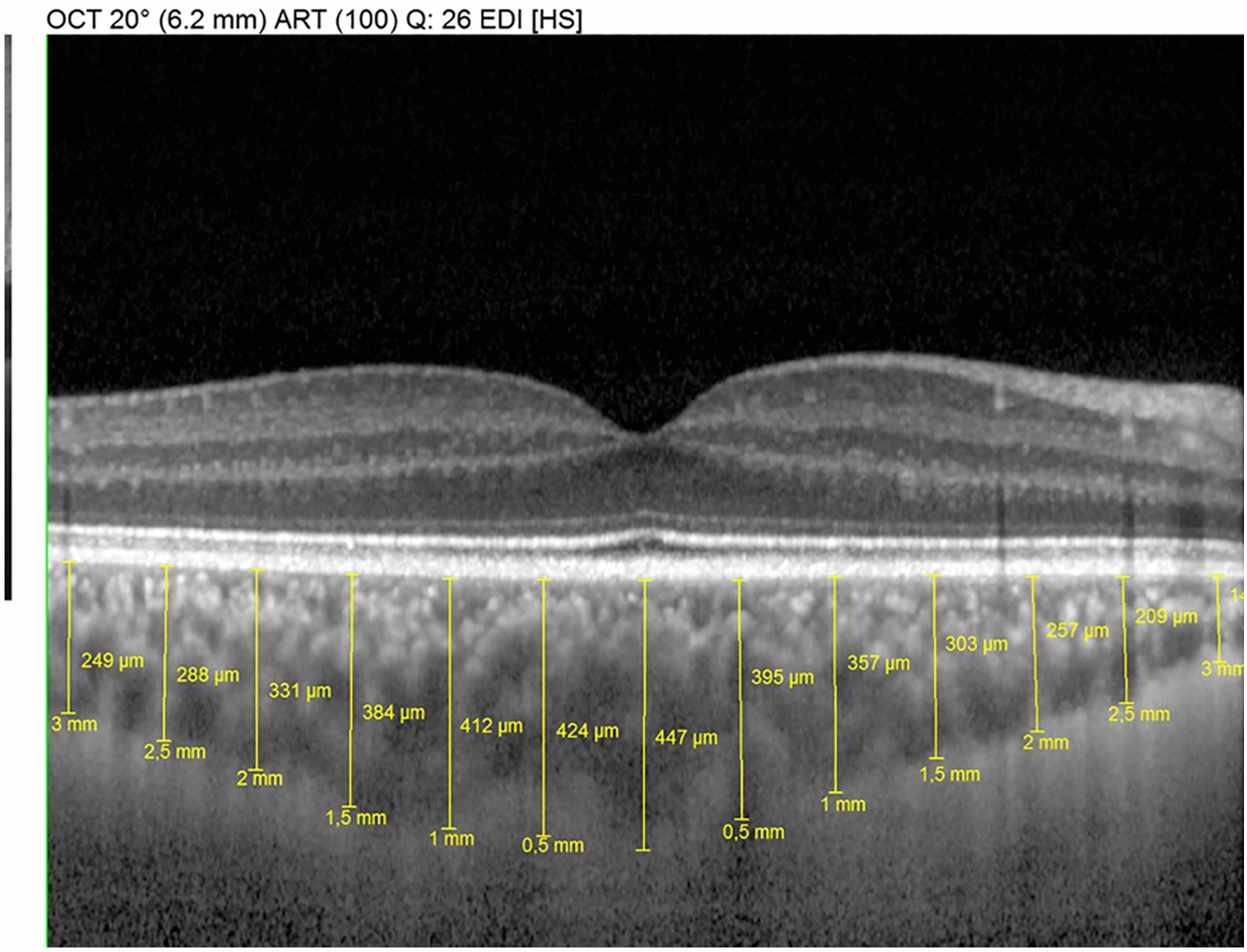
An EDI-OCT scan from one study patient. The choroidal thickness was measured perpendicularly from the outer edge of the retinal pigment epithelium to the chorioscleral boundary at five points: subfoveal, nasal perifoveal (500 µm nasal to the fovea), nasal perifoveal (1000 µm nasal to the fovea), temporal perifoveal (500 μm temporal to the fovea), and temporal parafoveal (1000 μm temporal to the fovea)

The horizontal and vertical enhanced-depth imaging EDI-OCT crosshair scans centered on the fovea were analyzed. Choroidal imaging was performed as a single line scan, 7.2 mm long, passing directly through the foveal center, using the EDI protocol in the Heidelberg machine. The choroidal thickness was measured perpendicularly from the outer edge of the retinal pigment epithelium to the chorioscleral boundary at five points: subfoveal, nasal perifoveal (500 µm nasal to the fovea), nasal perifoveal (1000 µm nasal to the fovea), temporal perifoveal (500 μm temporal to the fovea), and temporal parafoveal (1000 μm temporal to the fovea) (Fig. 1).

Statistical analysis was performed using the Stata v.14.0 software (Stata Corp., College Station, TX, USA). Frequency tables were used for descriptive analyses. The normality of continuous data was assessed using the Shapiro–Wilk test. The groups were compared in terms of demographic characteristics using the Chi-Square test for categorical variables and the Mann–Whitney test for not normally distributed continuous variables. Considering that individuals contributed with measurements of both eyes, the retinal and choroidal thickness values of those with SCD and controls were compared by Generalized Estimating Equations (GEE) models. P < 0.05 was considered statistically significant.

## Results

The study group included 79 eyes of 40 subjects (20 boys, 20 girls; mean age, 10.05 ± 3.37 years). Thirty-six eyes of 19 healthy patients comprised the control group. The mean VA was 20/20, and the mean refractive error was 0.50 D. Hemoglobin SS was observed in 33 patients (82%), SC in six patients (15%), and S-beta thalassemia in one patient (3%). Among the 40 patients with SCD, retinal findings were observed in 26 eyes (32.9%), i.e., retinal tortuosity in 20 eyes (25.3%), retinal arteriolar narrowing in five eyes (6.3%) and a black sunburst in one eye (1.27%). No study patients had proliferative SC retinopathy. Table 1 shows the demographic characteristics of the patients in each group.

**Table 1:** Baseline demographic characteristics of children with SCD and control subjects

	SCD	Control	p value
Sex, no. (%)			
Male	20 (50%)	10 (52.63%)	0.850
Female	20 (50%)	9 (47.37%)	
Age, mean age ± standard deviation (median)	10.05 ± 3.37 (9.50)	10.53 ± 3.82 (9.00)	0.7134

**Fig. 2:**
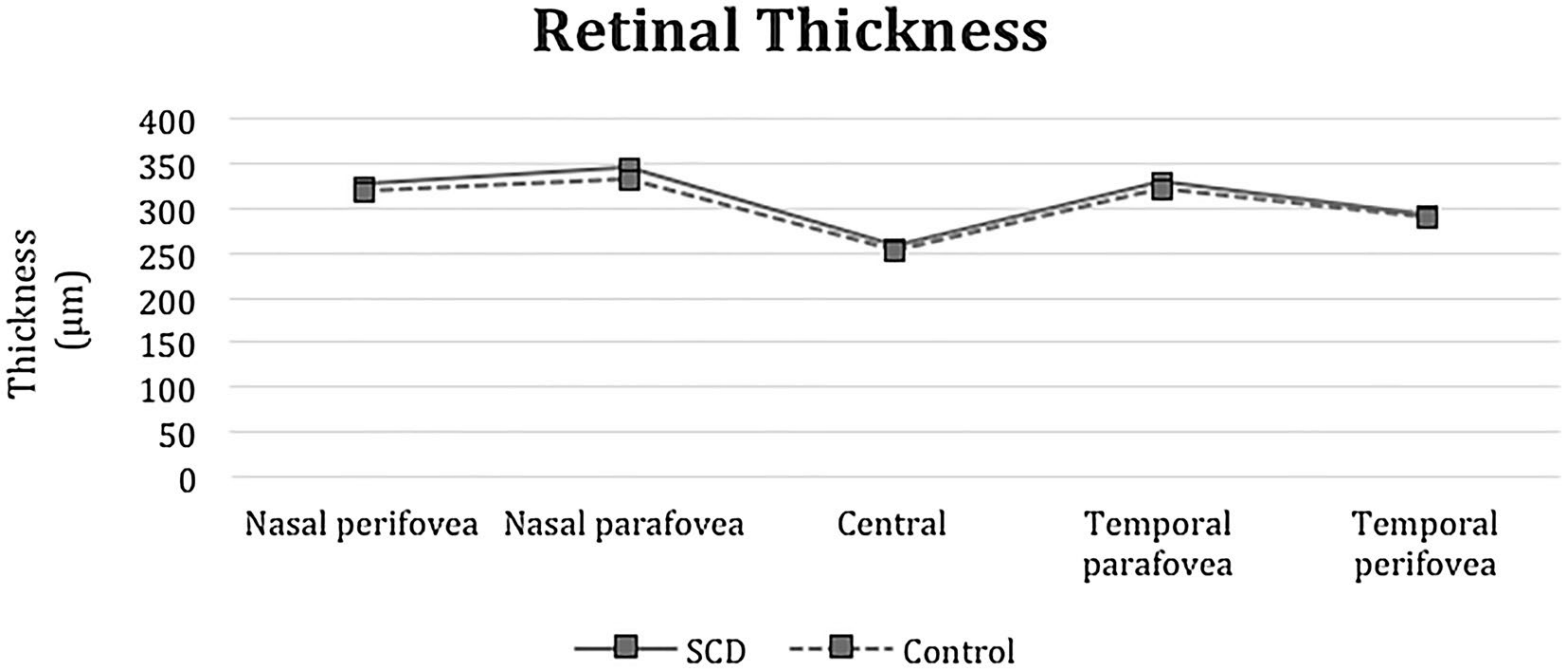
Comparison of retinal thickness subfield measurements between the SCD and control groups. The subfoveal choroidal thickness is 240.78 ± 21.27, and the mean foveal retinal thickness is 256.39 ± 20.84. There is no significant reduction in the retinal thickness in the SCD group compared with the control eyes

**Fig. 3:**
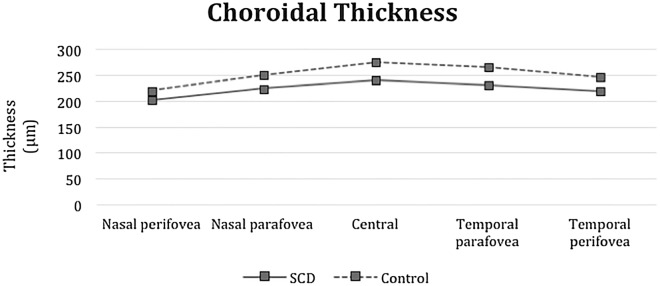
Comparison of choroidal thickness measurements between the SCD and control groups. The choroidal thickness measurements decreased in the SCD eyes. Compared with the control eyes, the choroidal thickness measurements in the SCD eyes are decreased in the subfovea parafovea (p < 0.001) and temporal parafovea (p < 0.001), nasal parafovea (p < 0.01), temporal perifovea (p < 0.01), and nasal perifovea (p =  < 0.01)

The subfoveal choroidal thickness was 240.78 ± 21.27, and the mean foveal retinal thickness was 256.39 ± 20.84. Despite the observation of two patients with retinal thinning in the sickle cell anemia group, we found a statistically significant increase in retinal thickness in the nasal parafovea (p = 0.026), inferior parafovea (p = 0.011), temporal parafovea (p = 0.036) in the sickle cell anemia group compared to the control group. (Table 2, Fig. 2). The choroidal thickness measurements decreased in the SCD eyes. Compared with the control eyes, the choroidal thickness measurements in the SCD eyes decreased in the subfovea (p < 0.001), temporal parafovea (p < 0.001), nasal parafovea (p < 0.001), temporal perifovea (p < 0.001), and nasal perifovea (p < 0.001) (Table 2, Fig. 3).

**Table 2:** Comparison of SD-OCT retinal and choroidal thickness subfield measurements in the SCD and control groups

	SCD	Control	p value
	Mean µm ± standard deviation (median)	Mean µm ± standard deviation (median)	
Retinal thickness			
Central	256.39 ± 20.84 (256.00)	254.58 ± 18.89 (255.00)	0.748
Superior parafovea	344.65 ± 21.13 (344.50)	340.06 ± 18.80 (337.00)	0.378
Nasal parafovea	345.58 ± 17.21 (343.00)	334.00 ± 22.24 (330.00)	0.026*
Inferior parafovea	343.07 ± 14.80 (342.00)	332.28 ± 18.26 (331.50)	0.011*
Temporal parafovea	330.83 ± 16.02 (331.00)	322.14 ± 15.89 (320.00)	0.036*
Superior perifovea	317.73 ± 13.24 (316.00)	311.18 ± 21.20 (305.00)	0.285
Nasal perifovea	326.05 ± 15.06 (325.00)	319.75 ± 19.14 (318.50)	0.193
Inferior perifovea	309.41 ± 17.15 (307.00)	300.96 ± 17.18 (299.00)	0.075
Temporal perifovea	293.24 ± 11.09 (292.00)	291.60 ± 23.13 (286.00)	0.733
Choroidal thickness			
Subfovea	240.78 ± 21.27 (238.00)	275.87 ± 41.14 (262.50)	< 0.001*
Temporal parafovea	231.08 ± 21.01 (230.00)	266.16 ± 43.43 (255.00)	< 0.001*
Nasal parafovea	224.52 ± 17.97 (224.00)	251.44 ± 40.92 (236.00)	< 0.001*
Temporal perifovea	220.15 ± 30.47 (212.00)	247.28 ± 47.18 (228.00)	< 0.001*
Nasal perifovea	202.36 ± 17.53 (200.00)	220.69 ± 39.94 (214.50)	< 0.001*

^*^p < 0.05 is significant

When we analyzed the retinal and choroidal thickness values in the patients with SCD according to the status of the retinopathy, no significant retinal or choroidal thinning was seen in the subgroups (SCD with retinopathy vs. SCD without retinopathy) (Table 3).

**Table 3:** Comparison of retinal and choroidal thickness measurements using EDI-OCT in patients with SCD with and without retinopathy

	SCD with retinopathy Mean µm ± standard deviation (median)	SCD without retinopathy Mean µm ± standard deviation (median)	p value
Retinal thickness			
Central	253.00 ± 23.39 (256.00)	258.15 ± 19.40 (256.00)	0.665
Superior parafovea	347.48 ± 30.20 (346.00)	343.16 ± 14.37 (344.00)	0.423
Nasal parafovea	348.52 ± 22.39 (346.00)	344.06 ± 13.79 (343.00)	0.298
Inferior parafovea	345.26 ± 19.94 (344.00)	341.94 ± 11.33 (341.50)	0.313
Temporal parafovea	332.11 ± 18.60 (331.00)	330.17 ± 14.66 (329.00)	0.688
Superior perifovea	323.26 ± 13.12 (320.00)	314.84 ± 12.49 (314.50)	0.029*
Nasal perifovea	330.11 ± 18.58 (329.00)	323.94 ± 12.55 (325.00)	0.636
Inferior perifovea	313.36 ± 17.04 (313.00)	307.26 ± 17.00 (303.50)	0.356
Temporal perifovea	297.44 ± 11.97 (294.00)	291.06 ± 10.04 (289.00)	0.058
Choroidal thickness			
Fovea	237.50 ± 17.86 (235.50)	242.46 ± 22.85 (240.00)	0.391
Temporal parafovea	230.45 ± 17.18 (230.00)	231.41 ± 22.93 (229.00)	0.854
Nasal parafovea	224.40 ± 16.03 (225.00)	224.59 ± 19.09 (222.00)	0.979
Temporal perifovea	228.70 ± 34.87 (214.00)	215.77 ± 27.40 (210.00)	0.108
Nasal perifovea	202.80 ± 16.62 (199.00)	202.13 ± 18.18 (200.00)	0.953

*p < 0.05 is significant

## Discussion

Although findings of SC retinopathy are mostly present peripherally, macular changes due to vascular occlusion in SCD have been reported using fluorescein angiography (FA) [5, 11, 12] electroretinography [13], and histopathology. In the FA studies, the foveal avascular zone was enlarged in patients with SCD. Some studies have reported that macular infarcts can lead to low VA, low foveal sensitivity, and reduced retinal layer thickness on OCT images [5, 6, 14].

Previous SD-OCT studies in adults with SCD have suggested that areas of macular thinning occur secondary to retinal arteriolar occlusion [6, 7, 9]. More recent studies have reported subclinical central macular thinning, foveal splaying, and focal macular thinning in about 50% of eyes with SC hemoglobinopathies [6, 7]. The studies also suggested that central macular splaying/focal thinning may indicate ischemia attributable to vascular occlusions in the capillaries around the fovea.

Since the choroid is a highly vascularized tissue, it is possible that, as in the retina, a vessel occlusive phenomena can occur at the level of the choroid resulting from the irregularly shaped red cells. FA, indocyanine green angiography, and ultrasonography do not visualize the choroid completely. Swept-source OCT and EDI-OCT facilitate better visualization of the choroidal anatomy. This noninvasive tool is useful to evaluate the choroidal thickness and indirectly detect choroidal changes in pathologic states such as choroidal capillary non-perfusion due to subclinical embolic events [8]. Mathew et al. [8] reported significant choroidal thinning in adults with SCD in contrast to discrete areas of retinal thinning. The investigators did not find a correlation between choroidal thinning and areas of macular thinning and suggested that macular microarteriolar occlusions are independent of changes in the choroidal circulation [8].

In the current study, EDI-OCT showed that the macular choroidal thickness is thinner in pediatric patients with SCD. The choroidal thickness in these patients was not correlated with age and was not the normal choroidal thickness seen in the normal control eyes. However, the current study did not identify significant retinal thinning in the nine ETDRS subfields compared with the control group. Retinal thinning was seen in only two patients with SCD. Some authors have reported that the choriocapilar is more sensitive to the effects of hypoxic or ischemic diseases than other ocular components, which may be explained by the lobular arrangement and larger volume of the choroidal vasculature [8, 15]. Choroidal thinning can occur due to sickling of red blood cells and subsequent reduction in choriocapillar blood flow.

Reports that included both adults and children with SCD showed decreased macular thickness, which was more prominent in the temporal macula. A large series of adults with SCD reported a prevalence of 43% of eyes with discrete areas of macular thinning on fovea-centered SD-OCT [8]. Martin et al. reported areas of temporal retinal thinning in 38% of eyes and suggested that retinal thinning may occur early in the disease course [9]. Pahl et al. [10] evaluated 24 eyes of adolescents with SCD aged 10 to 19 years and reported macular thinning and flow abnormalities on OCT angiography (OCTA) images. In contrast, Ong et al. observed less dense vasculature on the OCTA images but with similar retinal thickness compared with an age- and race-matched control group and suggested that the microvascular abnormalities may precede the structural retinal thinning [16]. Unlike previous studies, our study showed an increase in retinal thickness in asymptomatic children with sickle cell anemia, which may suggest that retinal thinning occurs at a later stage of the disease.

Despite the normal retinal thickness in the study eyes, we found choroidal thinning in pediatric patients with SCD. Our results suggested that choroidal thinning may precede the retinal thinning in asymptomatic children with SCD, possibly due to the earlier sickling in the choroidal vessels as a result of slower vascular flow.

Ideally, SCD retinopathy should be diagnosed before the proliferative stage develops, since patients may experience visual loss due to macular microinfarcts during the non-proliferative stage. Because patients with SCD begin to exhibit evidence of proliferative retinopathy around 10 years of age, pediatric patients with SCD should be referred to an ophthalmologist as soon as possible [17].

The current study had some limitations. In addition to its retrospective design and small number of study eyes, the choroidal thickness measurements were performed manually. Another limitation is a single choroidal measurement for a constantly changing structure. We performed a single measurement because it is an exam that requires the child's cooperation. To minimize this effect of daily variation, we performed all exams between 9 and 11 am.

In conclusion, the current study showed that the choroid was significantly thinner in the macular area of pediatric patients with SCD, which may be related to the choroidal microvasculature changes that precede retinal thinning.

## Data Availability

The datasets used and/or analysed during the current study are available from the corresponding author on reasonable request.

## Abbreviations

SCD: Sickle cell disease
SD-OCT: Spectral-domain optical coherence tomography
VA: Visual acuity
D: Diopters
ETDRS: Early Treatment Diabetic Retinopathy Study
FA: Fluorescein angiography
EDI-OCT: Enhanced-depth imaging optical coherence tomography
OCTA: Optical coherence tomography angiography
